# Medical Association of Georgia

**Published:** 1894-04

**Authors:** 


### Editorial.

---

### MEDICAL ASSOCIATION OF GEORGIA.

The forty-fifth annual meeting of the Medical Association of Georgia will convene in this city on the 18th, 19th and 20th of April. That the meeting will be one of the most interesting in the history of the Association goes without saying. A glance at the appended programme will show that in number, as well as quality, the papers offered are of high merit and are in keeping with the rapid advancement in medicine and surgery.

It is encumbent upon every regular physician in Georgia to be enrolled upon the membership of this useful and time-honored organization. The binding together of the regular profession of the State into a great brotherhood, for such it is, or should be, encourages and promotes advancement in the science and art of our profession in a greater degree than by means of any other organization.

Local medical societies are institutions of great usefulness, and should be encouraged, but the number in this State is so small that only a limited number of the profession have the pleasure and privilege of participating in the transactions.

In the State Society the country practitioner and the city specialist meet, and the exchange of ideas and the discussions arising from the great number of interesting papers presented are always productive of great good.

The complete eradication of every semblance of politics and the notable absence of any evidence of "wire-pulling" and "slates" make glad the man who very sensibly advocates the theory that politics and medicine should be widely separated.

The profession of Atlanta extends a cordial invitation to every member of the profession in Georgia, and those who come can be assured a pleasant sojourn in our beautiful city.

Treatment of Pneumonia, W. C. Humphries, M. D., Acworth.

Fluid Extract Jaborandi as an Abortive Treatment in Pneumonia, O. H. Buford, M.D., Cartersville.

Pathology and Treatment of Pneumonia, J. C. Johnson, M.D., Atlanta.

Castration for Crime, J. G. Hopkins, M.D., Thomasville.

A Case of Sarcoma of the Ileum following a Railway Injury, H. J. Williams, M.D., Macon.

Operation for Fibro-Cystic Sarcoma Involving the Right Inferior Maxillary Bone, J. McFadden Gaston, M.D., Atlanta.

Malignant Growths of the Skin; Their Diagnosis and Treatment, M. B. Hutchins, M.D., Atlanta.

Suggestion and its Therapeutic Uses, Samuel C. Benedict, M.D., Athens.

Treatment of Corneal Ulcers with the Galvano-cautery, A. G. Hobbs, M.D., Atlanta.

Chronic Sore Legs and How to Cure Them, J. W. Hallum, M.D., Carrollton.

Diagnosis, H. Perdue, M.D., Barnesville.

A Case of Gonorrhea in a Female, R. J. Nunn, M.D., Savannah.

The Treatment of Typhoid Fever, M. A. Clark, M.D., Barnesville.

Appendicitis, with Report of Cases, Floyd W. McRae, M.D., Atlanta.

Some Remarks upon Appendicitis and Report of Cases, W. S. Elkin, M.D., Atlanta.

The Termination of Appendicitis when not Subjected to Operation, A. M. Cartiledge, M.D., Louisville, Ky.

Foreign Bodies in Larynx, J. M. Hull, M.D., Augusta.

Report of Case of Inflammation of External Auditory Canal, Following Facial Erysipelas, P. R. Cortelyou, M.D., Marietta.

Otorrhea, Dunbar Roy, M.D., Atlanta.

Orator's Address at twelve o'clock, Sam'l C. Benedict, M. D., Athens.

Teething, H. McHatton, M.D., Macon.

Treatment of Surgical Shock, W. F. Westmoreland, M.D., Atlanta.

An Operation for Hemorrhoids, R. H. Taylor, M.D., Griffin.

The Time for Operation in Strangulated Hernia, W. P. Nicolson, M.D., Atlanta.

Primary and Diphtheritic Croup, the Operative and the Expectant or Medical Treatment, F. M. Ridley, M. D., LaGrange.

Drainage of the Peritoneal Cavity, The Use of the Syphon Pump, W. B. Gilmer, M.D., Macon.

Phlegmasia Alba Dolens, Geo. H. Noble, M.D., Atlanta.

Cancer of the Uterus, the Remote Result of Operative Interference, W. A. Crow, M.D., Atlanta.

Sacrificial Surgery of Ovaries, Tubes and Uterus, R. P. Cox, M.D., Rome.

Rare Cases in Obstetrics, W. F. Holt, M.D., Macon.

Trephining in Head Injuries with Paralysis of Opposite Arm, followed by Fungus Cerebri, R. M. Harbin, M.D., Calhoun.

The Extraction of Clear Lenses—Report of Five Cases, H. E. Stafford, M.D., New York.

A Cataract, J. H. Shorter, M.D., Macon.

Conjunctival Cysts in Each Eye, W. L. Bullard, M.D., Columbus.

Nasal Sprays—What to Use, Geo. W. Brown, M.D., Atlanta.

Typhoid and Typho-Malarial Fevers, Their Differential Characteristic Features, A. A. Smith, M.D., Hawkinsville.

Case Report, Extra-Uterine Superfetation, R. R. Kime, M.D., Atlanta.

A Case of Extra-Uterine Pregnancy, J. G. Earnest, M.D., Atlanta.

Treatment of Traumatic Epilepsy, with Report of Case, J. I. Darby, M.D., Americus.

Galvanism in Office Practice and in Gynecology, L. C. LeHardy, M.D., Savannah.

The Tampon in Gynecology, J. C. Avary, M.D., Atlanta.

A Plea for the Closer Recognition of Dermatology as a Specialty, Bernard Wolff, M.D., Atlanta.

The General Practitioner Versus Gynecology Preventive and Non-Preventive, R. R. Kime, M. D., Atlanta.

Medical Legislation, A. C. Blain, M.D., Macon.

LaGrippe—Some Unusual Sequences—Cases, J. M. Head, M.D., Zebulon.

Treatment of Compound Fracture of the Skull, W. S. Goldsmith, M.D., Atlanta.

The Recent Outbreak of Smallpox in Atlanta, J. C. Avary, M.D., Atlanta.

Suture of the Tendo Achillis, Report of Case and Exhibition of Patient, L. B. Grandy, M.D., Atlanta.

Endometritis, Cervical and Corporal; Pathology, Symptomatology and Treatment, W. W. Stewart, M.D., Columbus.

